# Real-world Evidence for the Treatment of Rosacea with Sulfur or Metronidazole Preparation in Japanese Patients

**DOI:** 10.31662/jmaj.2023-0100

**Published:** 2023-09-20

**Authors:** Yoshimasa Nobeyama, Yoshiko Aihara, Akihiko Asahina

**Affiliations:** 1 Ai Dermatology Clinic, Utsunomiya, Japan; 2 Department of Dermatology, The Jikei University School of Medicine, Tokyo, Japan

**Keywords:** topical treatment, sulfur, metronidazole, erythematotelangiectatic rosacea, papulopustular rosacea

## Abstract

**Introduction:**

There remains to be lacking real-world evidence for the treatment of rosacea with a topical sulfur preparation (TSP) or topical metronidazole preparation (TMP) among Japanese patients. Therefore, in this study, we examined the effects of TSP and TMP on rosacea in Japanese patients in real-world clinical settings.

**Methods:**

This retrospective observational analysis reviewed the medical records of 47 Japanese patients who were treated with TSP or TMP for more than 8 weeks in our clinic. Disease severity was evaluated using the Investigator Global Assessment (IGA) and the visual analog scale (VAS) for itching, burning sensation, flushing, and hypersensitivity before and 8 weeks after the initiation of the intervention.

**Results:**

In total, 10 erythematotelangiectatic rosacea (ETR) and 12 papulopustular rosacea (PPR) patients treated with TSP and 12 ETR and 13 PPR patients treated with TMP were analyzed. IGA and VAS scores for itching, burning sensation, flushing, and hypersensitivity were noted to significantly improve in the ETR and PPR patient groups treated with TSP and both groups treated with TMP, except for the VAS score for itching in the TSP-treated ETR group. No significant differences were observed in terms of the improvement rates of IGA, VAS scores, or the prevalence of adverse events between the TSP- and TMP-treated groups.

**Conclusions:**

As per our findings, TSP and TMP have similarly favorable effects on both ETR and PPR in Japanese patients in real-world settings.

## Introduction

Rosacea is a common cutaneous disease characterized by chronic inflammation; it mainly affects the cheeks, nose, and forehead ^(1)^. The National Rosacea Society (NRS) Expert Committee in 2002 classified rosacea into the following subtypes: (i) erythematotelangiectatic rosacea (ETR), (ii) papulopustular rosacea (PPR), (iii) phymatous rosacea, and iv) ocular rosacea ^(2)^. However, this classification was not adopted by the global ROSacea COnsensus (ROSCO) panel, a conference organized in 2016 ^(3)^. According to this classification, many therapeutic options have been evaluated. Currently, there are a number of available therapeutic options for rosacea including oral medicines, injective agents, electromagnetic wave-based treatments, and topical agents ^(4)^. Among them, topical agents have a number of merits, including a lower risk of systemic adverse events than systemic treatments and lower costs than electromagnetic wave-based treatments and treatments with injective agents. Therefore, topical agents are deemed essential for the treatment of rosacea.

A few studies in Western countries concurrently indicated the similar effectiveness of a topical sulfur preparation (TSP) and topical metronidazole preparation (TMP) for rosacea. In fact, Torok et al. reported a complete, excellent, or good improvement in 51 out of 75 patients treated with 10% sulfacetamide plus 5% sulfur cream and in 43 out of 77 patients treated with 0.75% metronidazole cream ^(5)^. Moreover, in a study by Lebwohl et al. wherein 63 patients with rosacea were examined, it was determined that the effects of 10% sulfacetamide plus 5% sulfur lotion were similar to those of 0.75% metronidazole gel ^(6)^. On the other hand, racial differences in the development of rosacea have been suggested in real-world clinical settings. Rosacea appears to be less frequent in patients with darker skin tones ^(7)^. Furthermore, the subtype distribution of rosacea in Japanese individuals significantly differs from that in Caucasian individuals ^(8)^; the former rarely develop the phymatous type of rosacea, in contrast to the latter. In that context, data on responses to treatments for rosacea are thus required for each racial population.

In Japan, sulfur and camphor lotion^Ⓡ^ (60 mg sulfur and 5 mg dl-camphor per 1 mL; TSP) was the only topical agent approved for the treatment of rosacea by the Ministry of Health, Labour and Welfare by April 2022. In May 2022, Rozex Gel^Ⓡ^ (metronidazole 7.5 mg per 1g; TMP) was approved as a topical agent based on the findings of a randomized, vehicle-controlled, phase 3 study on 130 Japanese patients showing the favorable effects of TMP (Rozex Gel^Ⓡ^) for rosacea ^(9)^. Rosacea patients with an Investigator Global Assessment (IGA) score of ≥3 and an inflammatory lesion count (papules/pustules) of ≥11 and ≤ 40 on the whole face were enrolled in this phase 3 study; therefore, the conditions of the participating rosacea patients were restricted.

Dermatologists can now select TSP or TMP as a topical agent for the treatment of rosacea in Japan. However, there is currently very limited real-world evidence for the efficacy and safety of TSP for both ETR and PPR and TMP for ETR, particularly in Japanese patients. Therefore, in this study, we aim to obtain real-world evidence for the treatment of rosacea by TSP and TMP in Japanese patients.

## Materials and Methods

### Patients

This retrospective observational study was approved by the Ethics Committee of the Jikei University School of Medicine (approval code: 33-223). Informed consent was obtained in an opt-out form on the website. In total, 47 Japanese patients were analyzed after meeting the following criteria (Supplementary Table 1): (i) referral to the Ai Dermatology Clinic between June 2022 and October 2022; (ii) fulfillment of the diagnostic criteria for rosacea as defined in the “Diagnostic criteria” subsection; (iii) receiving the treatment with TSP or TMP for more than 8 weeks in our clinic; (iv) evaluated with IGA scores, visual analog scale (VAS) scores for itching, burning sensation, flushing, and hypersensitivity, and a *Demodex* mite examination before and 8 weeks after the initiation of the treatment intervention; and (v) not receiving systemic treatments within at least 6 weeks immediately before the assessments. Meanwhile, the exclusion criteria are as follows: (i) ongoing pregnancy; (ii) receiving topical or systemic treatment for any disease at the first visit; and (iii) having systemic disease potentially affecting systemic or cutaneous manifestations at the first visit. Clinical manifestations were evaluated by at least two Japanese Dermatological Association-certified dermatologists.

### Diagnostic criteria

Rosacea was diagnosed according to the criteria of the ROSCO panel, namely, facial lesions presenting with more than one of the following criteria: (i) phymatous changes and (ii) persistent centrofacial erythema associated with periodic intensification by potential triggering factors ^(3)^. Other inflammatory facial diseases, such as seborrheic dermatitis, acne vulgaris, malar rash of lupus erythematosus, and contact dermatitis, were excluded.

The clinical subtypes of rosacea were classified according to the subtype classification of NRS in 2002: (i) ETR was defined as facial lesions presenting with flushing and persistent central facial erythema with or without telangiectasia, (ii) PPR was defined as facial lesions presenting with persistent central facial erythema with transient central facial papules, pustules, or both, (iii) phymatous rosacea was defined as facial lesions presenting with thickening skin and irregular surface nodules, and (iv) ocular rosacea was defined as rosacea-associated ophthalmologic lesions, including blepharoconjunctivitis with eyelid margin inflammation, meibomian gland dysfunction, and corneal complications, such as vascularization, ulceration, scarring, and perforation ^(2), (10)^. However, ocular rosacea was not examined, as this present study has focused on the topical treatments for cutaneous manifestations.

We considered rosacea with one or more papules/pustules and without phymatous changes to be PPR. Meanwhile, rosacea without both papules/pustules and phymatous changes was regarded as ETR. These subtypes were identified exclusively by the criteria of NRS (2002) at entry. Therefore, subtypes did not overlap in the patients examined in this study. We have observed facial lesions macroscopically and dermoscopically on cleaned skin.

Adverse events due to TSP and TMP were defined as any cutaneous and systemic manifestations which occurred after the initiation of the topical intervention.

### Assessment of severity

The IGA score was recorded based on a 5-point scale from 0 (clear; no inflammatory lesions and no erythema) to 4 (severe; many small to large papules and pustules or severe erythema) ^(11)^. The effects of the treatment intervention on rosacea were assessed as follows: the improvement value for VAS scores was defined as the value of the pre-intervention VAS score − the post-intervention VAS score; the improvement rate of IGA scores was defined as a value of (1 − post-intervention IGA score/pre-intervention IGA score) × 100 (%). Patients recorded a VAS score between 0 and 100 with a measure ranging from 0 to 100 mm. A VAS score of 100 was defined as the worst condition and that of 0 as no symptoms.

### *Demodex* mite examination

Scales obtained by brushing the affected cutaneous regions and/or contents obtained by extruding follicular pustules were treated with potassium hydroxide, and samples were thereafter examined under a microscope. The result of the examination was considered to be positive when more than one *Demodex* mite was detected.

### Statistical analysis

Statistical analyses were performed using Statistical Package for the Social Sciences version 22 software (IBM, Armonk, NY). Friedman test was conducted to examine quantitative differences, whereas Pearson’s chi-squared test was used to assess qualitative differences. The *p* values of <0.05 were considered to be significant.

## Results

### Effects on ETR and PPR in TSP- and TMP-treated groups

Among the 47 patients whose relevant data were mentioned on their medical records, 22 (46.8%), 25 (53.2%), and 0 (0.0%) patients were diagnosed with ETR, PPR, and phymatous rosacea, respectively, before the initiation of the therapeutic intervention (Supplementary Table 1). In total, 22 patients treated with TSP, including 10 ETR and 12 PPR patients, and 25 patients treated with TMP, including 12 ETR and 13 PPR patients, were assessed in this study (Table 1).

**Table 1.** Comparison of Patients’ Profiles between TSP and TMP before the Intervention.

	Topical treatment		
Parameters	TSP	TMP	*p*-value
Age (years)	46.2 ± 21.3	48.1 ± 11.9	0.469
Sex (number of patients)			0.237
Male	3	1	
Female	19	24	
Type of rosacea (number of patients)			0.861
ETR	10	12	
PPR	12	13
Results of *Demodex* exam. (number of patients)			0.632
Positive	18	20	
Negative	4	4	
Not examined	0	1	
IGA score (number of patients)			0.600
2	5	9	
3	9	9	
4	8	7	
VAS score (mean value ± standard deviation)			
Itch	50.2 ± 31.4	37.2 ± 25.7	0.094
Burning sensation	24.5 ± 28.8	29.1 ± 26.2	0.303
Flushing	27.1 ± 27.6	28.1 ± 27.5	0.839
Hypersensitivity	50.4 ± 29.9	54.6 ± 28.9	0.654
IGA score (mean value ± standard deviation)	3.14 ± 0.77	2.92 ± 0.81	0.353

**Figure 1.**
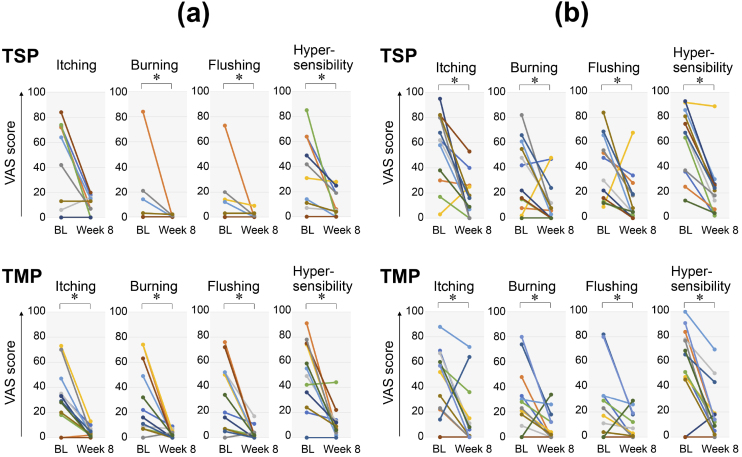
Changes in VAS scores in rosacea patients treated with TSP and TMP Values examined before and 8 weeks after the initiation of the therapeutic intervention for each patient are plotted. The points indicating values in each patient are connected by a straight line on the graph. Star marks indicate significant differences. BL, baseline before the therapeutic intervention. a) Line graph for patients with ETR. b) Line graph for patients with PPR.

**Figure 2.**
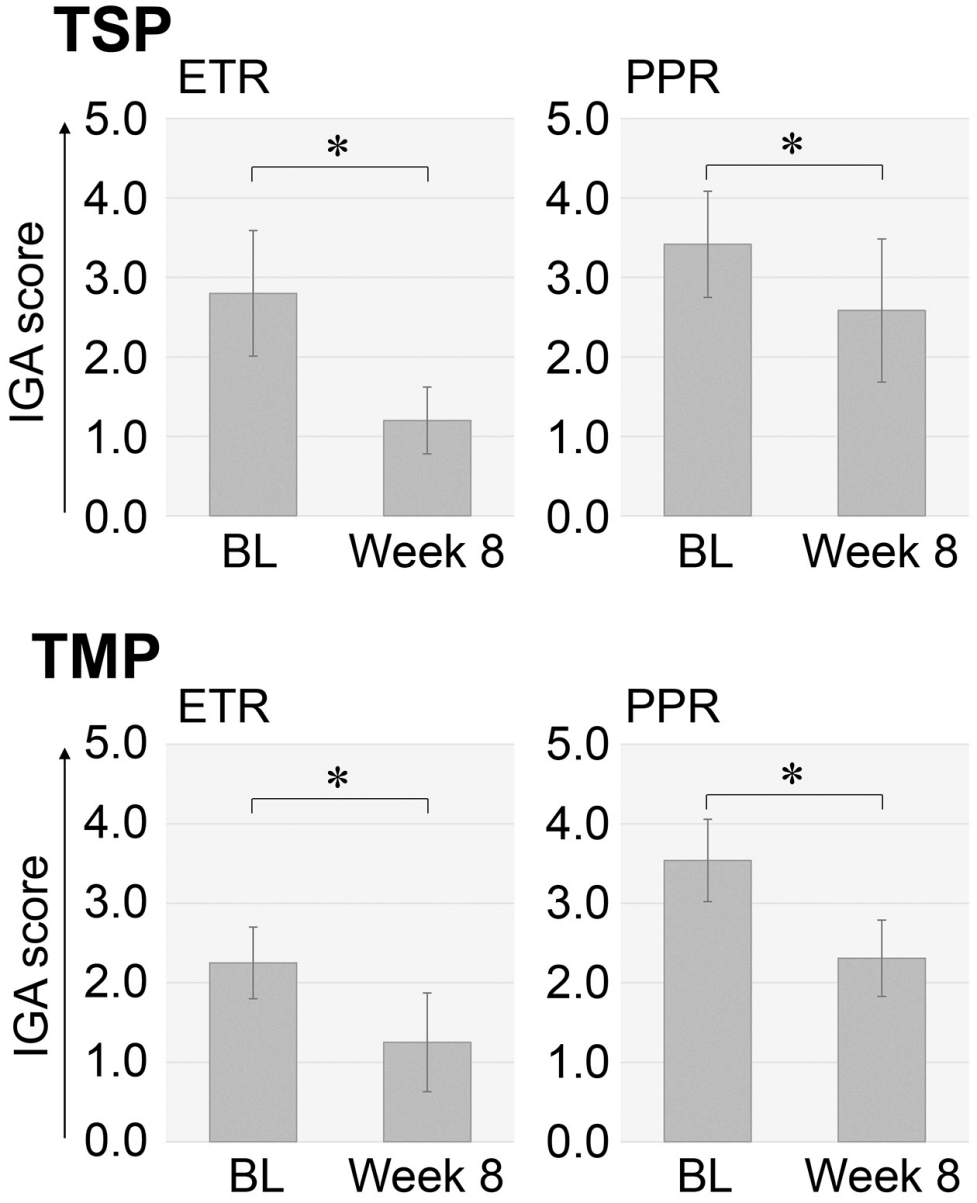
Changes in IGA scores in rosacea patients treated with TSP and TMP Mean values with standard deviations for IGA scores recorded before and 8 weeks after the initiation of the therapeutic intervention for each patient are presented as bar graphs. Star marks indicate significant differences. BL, baseline before the therapeutic intervention.

In ETR patients treated with TSP, IGA scores and VAS scores for burning sensation, flushing, and hypersensitivity were significantly lower than the baseline scores, which were recorded immediately before the initiation of the intervention (Figure 1a and 2). In ETR patients treated with TMP, IGA scores and VAS scores for itching, burning sensation, flushing, and hypersensitivity were also noted to be significantly lower than the baseline scores (Figure 1a and 2).

In PPR patients treated with TSP, IGA scores and VAS scores for itching, burning sensation, flushing, and hypersensitivity were significantly lower than the baseline scores (Figure 1b and 2). In PPR patients treated with TMP, IGA scores and VAS scores for itching, burning sensation, flushing, and hypersensitivity were significantly lower than the baseline scores (Figure 1b and 2).

Among the 22 TSP-treated patients who underwent the *Demodex* mite examination, the result was positive in 18 patients (81.2%) (Table 1). Eight weeks after the initiation of the intervention, the positivity rate has significantly decreased to 6 from the initial 18 positive patients (28.6%) (*p* < 0.001, Pearson’s chi-squared test) (Table 2). On the other hand, among the 24 TMP-treated patients who underwent the *Demodex* mite examination, the result was positive in 20 patients (83.3%) (Table 1). Eight weeks after the initiation of the intervention, the positivity rate significantly decreased to 8/20 patients (40.0%) (*p* = 0.003, Pearson’s chi-squared test) (Table 2).

**Table 2.** Comparison of Outcomes between TSP and TMP.

	Topical treatment		
Parameters	TSP	TMP	*p*-value
Results of *Demodex* exam. (number of patients)			0.212
Positive	6	8	
Negative	15	12	
Not examined	1	5	
Adverse events (number of patients)			0.215
Absent	13	19	
Present	9	6	

### Comparison of patients’ profiles between TSP- and TMP-treated groups

Before the initiation of the intervention, the distributions of age, sex, and rosacea subtypes did not significantly differ between the TSP- and TMP-treated groups (Table 1). Furthermore, IGA scores; VAS scores for itching, burning sensation, flushing, and hypersensitivity; and positivity rates in the *Demodex* mite examination were not significantly different. Based on these results, the TSP- and TMP-treated groups were considered to be comparable.

### Comparison of effects between TSP- and TMP-treated groups

Between the TSP- and TMP-treated groups, the decrease in the number of patients with a positive result in the *Demodex* mite examination did not significantly differ (Table 2). Moreover, no significant differences were observed in terms of the improvement rate in IGA scores or decreases in VAS scores for itching, burning sensation, flushing, and hypersensitivity (Supplementary Figure 1).

### Comparison of adverse events between TSP- and TMP-treated groups

During the 8-week study period, adverse events were reported, that is, contact dermatitis or an unpleasant sensation in 9/22 (40.9%) and 6/25 (24.0%) of TSP- and TMP-treated patients, respectively (Table 2). No significant difference was noted in terms of the prevalence of adverse events including contact dermatitis and an unpleasant sensation. Among the adverse events reported, contact dermatitis developed in seven TSP- and two TMP-treated patients (31.8% and 8.0%, respectively); contact dermatitis appeared more frequently in TSP-treated patients than TMP-treated patients (*p* = 0.038, Pearson’s chi-squared test). Unpleasant sensation developed in two TSP- and four TMP-treated patients (9.1% and 16.0%, respectively) with no significant difference between them.

## Discussion

In this present study, we aimed to provide real-world evidence for the treatment of rosacea using TSP and TMP in Japanese patients. As per our results, TSP and TMP have similarly favorable effects for both ETR and PPR in Japanese patients.

The basic patient profiles in our study have been noted to be similar to those in the phase 3 study conducted by Miyachi et al. that examined a patient group with a mean age of 43.8 years and a sex distribution of 23 male and 107 female patients ^(9)^. However, our study has included 14 patients with an IGA score of 2 before the initiation of the intervention, whereas Miyachi et al. have excluded these patients. Furthermore, our study included 22 patients with ETR, whereas the abovementioned phase 3 study did not because the latter’s inclusion criterion was ≥11 papules/pustules. We initially suggested that TMP was also effective for ETR in Japanese patients, which was eventually determined to be true as per our results.

Limited information is available on the effects of TSP on rosacea, particularly in Japanese patients. Therefore, some dermatologists have avoided using TSP to treat rosacea in Japanese patients, even though TSP was the only topical agent approved for the treatment of rosacea by the Japanese Ministry of Health, Labour and Welfare until April 2022. However, this present study clearly showed that TSP is significantly effective for ETR as well as PPR in Japanese patients. These results include the effects of the treatment for adverse events to clinical outcomes in real-world settings, although the effect of treatment for adverse events by itself was not evaluated in this present study. These results may be regarded as objective evidence for the favorable effects of TSP on ETR and PPR, thus encouraging dermatologists to use TSP as well as TMP to treat rosacea in Japanese patients.

This present study failed to demonstrate a significant improvement of itching in the ETR patient group treated with TSP unlike in the ETR patient group treated with TMP. On the other hand, this direct comparison showed no significant differences in terms of the improvement rates of clinical parameters including itching between TSP and TMP. Also, no significant difference was noted in terms of the prevalence of adverse events including contact dermatitis and an unpleasant sensation, although contact dermatitis by itself appeared more frequently in TSP-treated patients than in TMP-treated patients. Based on these findings, similar effectiveness and risks between TSP and TMP are suggested at least 8 weeks after the intervention. Therefore, the choice of treatment may depend on the preference of each patient; patients may be allowed to select a topical treatment with different drug formulations and odors. TSP may be suitable for patients with a preference for a lotion over a cream and acceptance of its characteristic odor. Similarly, TMP may be suitable for patients with a preference for a cream over a lotion and those who cannot tolerate the odor of TSP.

Many trials not only on TSP and TMP but also on the other topical agents for the treatment of rosacea have been conducted in Western countries ^(12)^. Taieb et al. reported that topical 1% ivermectin cream once a day was more effective than TMP (0.75%) twice a day ^(13)^. The effectiveness of topical minocycline, azelaic acid, and benzyl benzoate for PPR has also been reported ^(4), (14), (15)^. Topical oxymetazoline and brimonidine, adrenergic receptor agonists, were also shown to be effective for facial erythema associated with rosacea ^(16), (17)^. However, information on the effects of these agents in Japanese patients with rosacea remains to be very limited. Thus, the efficacy and safety of these topical agents for rosacea in Japanese patients also need to be examined in the near future.

This study has a few limitations that need to be addressed. First, this present study was conducted without placebo control, although we had no choice in real-world clinical settings. Second, this present study was a retrospective study. Therefore, bias may have been present in the selection of TSP or TMP for a rosacea patient by an attending physician. Third, the number of patients examined in this present study was small; therefore, more patients should be examined in future studies to obtain stronger evidence. Fourth, bias may have been present in the assessment of IGA scores because we evaluated the cutaneous manifestations of our patients before and 8 weeks after the initiation of the intervention. Fifth, the detailed data on the concomitant medications with TSP or TMP were not available in this present study. Such data might encourage a greater understanding of the results.

In conclusion, this present study provides real-world evidence for the treatment of rosacea with TSP or TMP in Japanese patients. The results obtained indicate the similarly favorable effects of TSP and TMP for both ETR and PPR in Japanese patients under real-world clinical settings. These present results will allow Japanese patients with rosacea to have better topical therapy options.

## Article Information

### Conflicts of Interest

Yoshimasa Nobeyama is a paid consultant/advisor of Maruho. Akihiko Asahina is a paid consultant/advisor of Maruho.

### Author Contributions

Y. N. designed and analyzed the data and wrote the manuscript.

Y. I. contributed to the concept and helped in the collection of the data.

A. A. is a supervisor and edited the manuscript.

All authors reviewed and approved the final manuscript.

### Approval by Institutional Review Board (IRB)

This study protocol was approved by the Ethics Committee of the Jikei University School of Medicine (approval code: 33-223).

### Data Availability Statement

All raw data are available in Supplementary Table 1

## Supplement

Supplementary Table 1

Supplementary Figure 1 Comparison of improvements between TSP and TMP
Mean values with standard deviations for improvement values in VAS scores and improvement rates in IGA scores are presented as bar graphs. The improvement value of VAS scores is defined as the value of the pre-intervention VAS score - the post-intervention VAS score. The improvement rate of IGA scores is defined as a value of (1 − post-intervention IGA score/pre-intervention IGA score) × 100 (%). Vertical and horizontal axes indicate topical therapeutic options and improvement values/rates, respectively. NS, not significantly different.
