# Supplementary material for: Real-world Evidence for the Treatment of Rosacea with Sulfur or Metronidazole Preparation in Japanese Patients
Source: JMA J. 2023 Sep 20;6(4):448–54. doi: 10.31662/jmaj.2023-0100 (PMC10628168; doi:10.31662/jmaj.2023-0100)
Supplement: Supplementary Table 1 [file 2433-3298-6-4-448-s001.pdf]

Supplementary Table 1. Raw data of the present study

Patient number	Age (years)	Sex	Type of rosacea	Topical treatment	VAS score for itching before intervention	VAS score for burning sensation before intervention	VAS score for flushing before intervention	VAS score for hypersensitivity before intervention	IGA score before intervention	Results of Demodex mite examination before intervention	VAS score for itching at 8 weeks	VAS score for burning sensation at 8 weeks	VAS score for flushing at 8 weeks	VAS score for hypersensitivity at 8 weeks	IGA score at 8 weeks	Results of Demodex mite examination at 8 weeks	Adverse events
1	71.4	female	PPR	TSP	62	42	48	37	4	positive	40	47	34	2	2	positive	absent
2	25.1	female	ETR	TSP	73	0	0	64	3	positive	18	0	0	19	1	negative	absent
3	19.6	female	PPR	TSP	30	8	52	25	4	positive	26	6	28	7	3	negative	absent
4	52.9	female	PPR	TSP	62	48	30	68	3	positive	16	12	4	14	3	positive	contact dermatitis
5	47.4	male	PPR	TSP	3	2	9	92	4	positive	25	48	68	89	4	positive	contact dermatitis
6	24.4	female	ETR	TSP	72	84	73	64	4	positive	7	0	0	6	2	negative	absent
7	35.5	female	PPR	TSP	58	61	66	86	4	positive	7	3	2	31	3	positive	absent
8	45.8	female	ETR	TSP	6	0	0	7	2	positive	16	0	0	5	1	negative	absent
9	71.3	female	ETR	TSP	0	0	14	31	2	positive	0	0	9	28	1	negative	contact dermatitis
10	31	female	PPR	TSP	17	15	13	64	4	positive	0	0	2	2	3	negative	contact dermatitis
11	33.8	female	PPR	TSP	95	22	22	93	3	positive	0	0	0	27	3	not examined	contact dermatitis
12	75.2	female	ETR	TSP	64	14	12	14	3	positive	15	0	0	0	2	negative	absent
13	72.3	female	ETR	TSP	74	0	0	85	2	positive	0	0	0	0	1	negative	absent
14	34.1	female	PPR	TSP	82	16	16	75	3	positive	53	0	0	26	3	positive	contact dermatitis
15	20.5	male	PPR	TSP	80	82	54	38	3	positive	0	8	18	18	1	negative	absent
16	22.3	female	PPR	TSP	82	55	84	81	2	negative	18	8	8	22	1	negative	absent
17	80.6	female	PPR	TSP	68	66	69	68	3	positive	16	24	19	24	2	positive	contact dermatitis
18	46.2	female	ETR	TSP	0	0	0	49	2	negative	0	0	0	25	1	negative	absent
19	83.3	female	ETR	TSP	84	0	0	0	3	positive	20	0	0	0	2	negative	absent
20	29.4	male	ETR	TSP	42	21	20	42	4	negative	7	1	1	19	3	negative	unpleasant sensation
21	34.5	female	ETR	TSP	13	3	3	11	3	negative	13	2	3	4	2	negative	unpleasant sensation
22	60.7	female	PPR	TSP	38	0	12	14	4	positive	9	0	5	4	3	negative	absent
23	55.6	female	ETR	TMP	35	9	52	49	3	positive	7	7	17	13	1	negative	absent
24	43.2	female	ETR	TMP	73	74	50	74	2	positive	13	5	1	6	1	positive	unpleasant sensation
25	56.1	female	ETR	TMP	47	49	52	55	2	positive	2	2	2	2	0	not examined	absent
26	23.2	female	PPR	TMP	67	9	11	78	4	positive	11	0	7	51	2	positive	absent
27	57.2	female	PPR	TMP	69	33	33	52	3	negative	6	0	0	5	2	negative	absent
28	69	female	ETR	TMP	18	7	7	42	2	positive	2	2	2	44	1	not examined	unpleasant sensation
29	52.1	female	ETR	TMP	34	22	20	20	3	negative	10	9	11	14	2	not examined	absent
30	46.8	female	ETR	TMP	33	16	16	36	2	positive	0	0	0	12	1	negative	absent
31	56.4	female	ETR	TMP	0	63	72	75	2	positive	0	0	2	22	2	negative	unpleasant sensation
32	48.1	female	ETR	TMP	0	74	76	91	2	negative	2	0	2	7	2	negative	absent
33	36.1	female	PPR	TMP	52	21	17	48	3	positive	15	4	3	19	2	positive	unpleasant sensation
34	68.3	female	ETR	TMP	70	0	0	78	2	positive	4	4	4	4	1	negative	absent
35	32	female	PPR	TMP	88	30	33	100	4	positive	72	26	26	70	2	positive	absent
36	26	female	PPR	TMP	57	28	29	52	3	positive	36	12	12	12	3	negative	absent
37	59.9	female	ETR	TMP	20	7	7	24	2	positive	3	0	0	9	1	positive	absent
38	44.7	female	PPR	TMP	0	0	0	0	3	positive	0	0	0	18	2	not examined	absent
39	32.9	female	ETR	TMP	29	11	5	0	2	positive	5	0	0	0	1	negative	absent
40	56.8	male	PPR	TMP	0	0	0	0	4	positive	0	0	0	0	2	negative	absent
41	46	female	ETR	TMP	28	32	34	59	3	positive	1	2	2	6	2	negative	absent
42	47.5	female	PPR	TMP	57	80	80	91	4	not examined	0	12	19	14	3	not examined	absent
43	44.8	female	PPR	TMP	23	23	23	77	4	positive	0	0	0	0	2	positive	absent
44	46.8	female	PPR	TMP	33	18	4	46	3	positive	1	2	1	2	2	negative	absent
45	54.2	female	PPR	TMP	14	74	82	66	4	positive	64	18	18	44	3	positive	contact dermatitis
46	39.8	female	PPR	TMP	60	0	0	69	4	positive	8	34	29	9	3	positive	contact dermatitis
47	59.1	female	PPR	TMP	22	48	0	84	3	negative	0	0	0	14	2	negative	absent
