# Supplementary material for: Real-world Evidence for the Treatment of Rosacea with Sulfur or Metronidazole Preparation in Japanese Patients
Source: JMA J. 2023 Sep 20;6(4):448–54. doi: 10.31662/jmaj.2023-0100 (PMC10628168; doi:10.31662/jmaj.2023-0100)

Supplementary Figure 1

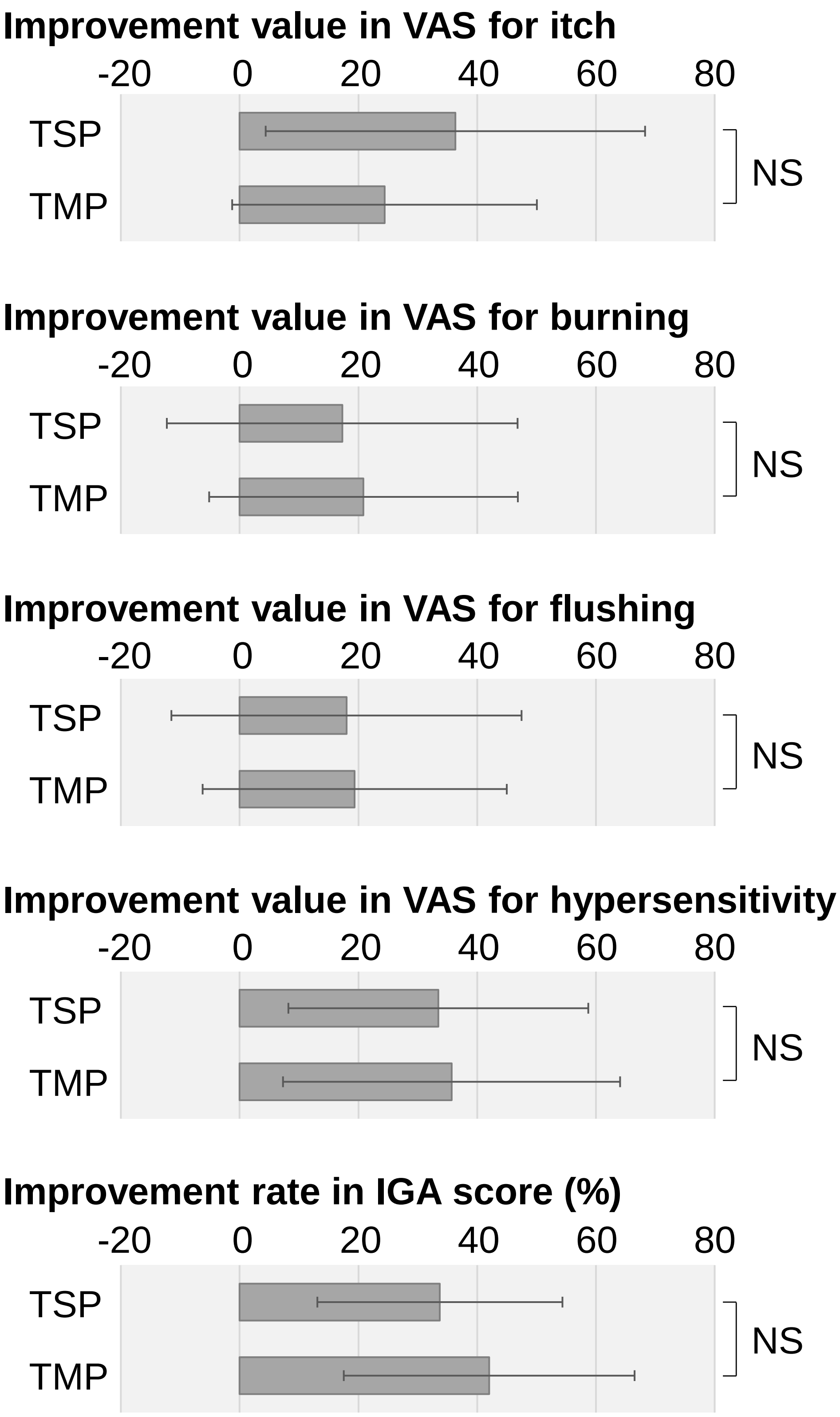

Supplement: Supplementary Figure 1 — Comparison of improvements between TSP and TMP Mean values with standard deviations for improvement values in VAS scores and improvement rates in IGA scores are presented as bar graphs. The improvement value of VAS scores is defined as the value of the pre-intervention VAS score - the post-intervention VAS score. The improvement rate of IGA scores is defined as a value of (1 − post-intervention IGA score/pre-intervention IGA score) × 100 (%). Vertical and horizontal axes indicate topical therapeutic options and improvement values/rates, respectively. NS, not significantly different. [file 2433-3298-6-4-448-s002.pdf]
